# Assessing biases of information contained in pedigrees for the classification of *BRCA*-genetic variants: a study arising from the ENIGMA analytical working group

**DOI:** 10.1186/s13053-016-0050-9

**Published:** 2016-04-30

**Authors:** C. H. H. Kerkhofs, A. B. Spurdle, P. J. Lindsey, D. E. Goldgar, E. B. Gómez-García

**Affiliations:** Department of Clinical Genetics, Maastricht University Medical Centre, Maastricht, The Netherlands; Department of Genetics and Computational Biology, QIMR Berghofer Medical Research Institute, Brisbane, Australia; Huntsman Cancer Institute and Department of Dermatology, University of Utah School of Medicine, Salt Lake City, USA

**Keywords:** BRCA1/2, Variant classification models, Family history, Variants of uncertain clinical significance, Intake pedigrees

## Abstract

**Purpose:**

One way of evaluating family history (FH) for classifying *BRCA1/2* variants of uncertain clinical significance (VUS) is to assess the “BRCA-ness” of a pedigree by comparing it to reference populations. The aim of this study was to assess if prediction of *BRCA* pathogenic variant (mutation) status based on pedigree information differed due to changes in FH since intake, both in families with a pathogenic variant (*BRCAm*) and in families with wild-type (*BRCAwt*).

**Patients and methods:**

We compared the *BRCA1/2* pathogenic variant detection probabilities between intake and most recent pedigree for *BRCAm* families (*n* = 64) and *BRCAwt* (*n* = 118) using the BRCAPRO software program.

**Results:**

Follow-up time between intake and most recent pedigree was significantly longer (*p* < 0.001) in the *BRCAm* compared to the *BRCAwt* families.

Among *BRCAwt* families, the probability to detect a pathogenic variant did not change over time. Conversely, among the *BRCAm*, this probability was significantly higher for most recent vs. intake pedigree (*p* = 0.006).

**Conclusion:**

Clinical scores change significantly over time for *BRCAm* families. This may be due to differences in follow-up, but also to differences in cancer risks from carrying a pathogenic variant in a highly penetrant gene. To reduce bias, models for VUS classification should incorporate FH collected at intake.

## Introduction

A significant fraction of *BRCA1/2* gene analyses result in the detection of a genetic variant for which the pathogenicity is unknown. These variants are named “variants of uncertain clinical significance” (VUS). VUS results occur in approximately 5–15 % of *BRCA1/2* sequencing tests with the likelihood dependent on the patient’s racial or ethnic background [1]. At the moment, ENIGMA (Evidence-based Network for the Interpretation of Germline Mutant Alleles; www.enigmaconsortium.org) has received >6000 submissions of *BRCA1/2* VUS corresponding to >3000 individual variants.

Different approaches have been suggested to determine the pathogenicity of VUS. Pedigree information is routinely used in the clinical setting to estimate the probability of finding a pathogenic variant in *BRCA1/2*, and thereby guide clinical testing. Hence pedigree information has utility to assess if a variant carrier portrays the features expected for an individual carrying a pathogenic variant (mutation) in *BRCA1/2*. Pathogenic variant probability scores can be obtained using existing software programs such as BOADICEA [2, 3], BRCAPRO [4, 5], Myriad [6, 7] or the Manchester scoring system [8] that measure how “BRCA-like” personal/family history is for a proband. Previously, we demonstrated that BRCAPRO scores can be used to predict the probability of a certain VUS being a pathogenic or a neutral variant in an analysis of a single cohort with defined selection criteria for *BRCA1/2* testing [9, 10]. Others have developed custom descriptions of family history, from a largely unselected patient group, for use in *BRCA* variant classification [11, 12].

A major advance in classifying VUS in *BRCA1/2* is the multifactorial probability-based model that was developed in 2004 [13], and has been extensively reviewed and updated ever since [11, 14]. Personal and cancer family history associated with the VUS is a strong component for this model [11]. An important question to address is the possibility of bias for pedigree information collected in the familial cancer setting, where records of family history change over time. Specifically in the families with a pathogenic variant, there is potential for more intensive follow-up and recording of family history due to cascade testing of relatives and ongoing surveillance of positive and at-risk family members. Since it is not standard practice in the Genetics centers to keep the intake pedigrees, such bias, if it exists, would have implications for the value of family history information for use in variant classification. The aim of our study was to establish if there are significant differences between mutation probability scores of intake and current pedigrees, if the extent of these differences is markedly altered between carriers of pathogenic variants versus non-carriers, and which parameters might account for such a difference. Our hypothesis was that, if calculations are restricted to the proband’s first and second degree relatives’ cancer history, information will not change significantly over time.

## Patients and methods

### Patients

#### Eligibility criteria

Our study population consisted of probands that consulted the department of Clinical Genetics of Maastricht University Medical Centre between 2009 and 2013 and underwent complete *BRCA1* and *BRCA2* analysis. Eligibility criteria remained the same in this period of time and were those used in the Netherlands for referral to a Genetics center [15]. All consecutive probands in whom a pathogenic variant was found were included for the positive control group. For the negative control group we collected twice as many probands, randomly selected by the last figure of the study number.

Intake pedigrees of all probands had been obtained and kept. Intake pedigrees are those made at the first proband’s visit containing information upon which decision to perform DNA-testing was made. The date of the intake pedigree and the date of the last update (i.e. the most recent pedigree) were annotated.

#### Clinical data

The probability to detect a pathogenic variant, measured as the BRCAPRO score, was our primary outcome measure. It was retrospectively obtained for all the included probands. The BRCAPRO model is distributed as a part of the counseling package CancerGene from U.T. Southwestern Medical Center at Dallas [3, 11]. BRCAPRO is a model that incorporates pathogenic variant frequencies and cancer-specific penetrances, in addition to clinical information about the proband and the first-degree and second-degree relatives, and with this estimates the probability of finding a pathogenic variant [4, 5]. For every proband, the BRCAPRO score based on the intake pedigree was obtained. If there had been any changes in the pedigree over time, the BRCAPRO score based on the most recent pedigree was also obtained.

### Laboratory diagnosis

*BRCA1* and *BRCA2* were analyzed from blood samples by denaturing high-performance liquid chromatography. Changes in denaturing high-performance liquid chromatography were verified by standard sequence analysis. To detect large duplications or deletions in *BRCA1*, multiplex ligation-dependent probe amplification analysis was also performed.

### Statistical analysis

Descriptive analysis was performed for the group with wt *BRCA* and the group with a pathogenic variant. The differences between the group with wt *BRCA* and the group with a pathogenic variant, and the differences between the intake and the most recent pedigree, were obtained with the *t* test for the continuous variables, the Poisson log-linear regression model for the discrete variables (e.g. number of patients with breast cancer), and the Fisher’s exact test for binary variables which were set up as two-by-two tables.

## Results

Sixty-four probands with a pathogenic variation in *BRCA*1 (*N* = 35) or *BRCA*2 (*N* = 29) and 118 probands with wt *BRCA1/2* were included. Follow-up time between the date of the intake pedigree and the date of the last update was significantly longer (*p* < 0.001) for families with a pathogenic variant compared to families with wt *BRCA* (580 days ± 364 vs. 127 days ± 153 respectively).

The clinical features of both groups are displayed in Table 1.

**Table 1:** Clinical features of families with breast and/or ovarian cancer: wild type (wt) vs. pathogenic variant (mutation; *BRCA*m)

	*BRCA* wt	*BRCA*m	
	*N* = 118	*N* = 64	
	Mean ± SD	Mean ± SD	*P*
Proband data			
BRCAPRO score total^a^			
Intake pedigree	0.281 ± 0.239	0.442 ± 0.306	<0.001
Recent pedigree	0.290 ± 0.242	0.505 ± 0.327	<0.001
Age of onset of first cancer^a^ (in years)	46.27 ± 11.695	43.84 ± 11.776	0.197
	Percentage	Percentage	
Sex (female)^b^	96.6	100	0.299
BC^b^	81.4	81.3	1.000
OC^b^	6.8	7.8	0.772
BC and OC^b^	3.4	6.2	0.455
Healthy^b^	8.5	4.7	0.548
Family data^c^	Mean^e^	Mean^e^	
No. of patients in each pedigree with			
BC^d^	2.31	2.34	0.883
OC^d^	0.28	0.31	0.730
BC and OC^d^	0.06	0.09	0.392
Bilateral BC^d^	0.42	0.42	0.985
BC in men^d^	0.04	0.06	0.551

^a^Univariate Gaussian linear regression model (*t*-test). ^b^Two-by-two table (Fisher’s exact test). ^c^Based on intake pedigree. ^d^Poisson log-linear regression model. ^e^Standard errors are in log scale, not shown

The BRCAPRO score, probability of *BRCA1/2* pathogenic variant detection, was significantly higher for the families with a pathogenic variant compared to families with wt *BRCA*: based both on the intake pedigree and on the most recent pedigree (*p* < 0.001). No significant differences were observed with regard to gender, age of onset of the first tumor, and type of cancer of the probands or in the families (Table 1).

Comparison of the pedigrees (intake versus recent) in families with a pathogenic variant (Table 2) showed that the BRCAPRO pathogenic variant detection probability was significantly higher (*p* = 0.006) when based on the most recent pedigree (0.505 ± 0.327) than on the intake pedigree (0.442 ± 0.306).

**Table 2:** Clinical features from the intake versus most recent pedigrees in families with *BRCA*wt and in families with *BRCA*m

*BRCA* genotype families	*BRCA* wild type (*n* = 118)			*BRCA* pathogenic variant (mutation) (*n* = 64)		
	Intake pedigree	Recent pedigree		Intake pedigree	Recent pedigree	
	Mean ± SD	Mean ± SD	*p*	Mean ± SD	Mean ± SD	*p*
BRCAPRO score total^a^	0.281 ± 0.239	0.290 ± 0.242	0.339	0.442 ± 0.306	0.505 ± 0.327	0.006
BRCAPRO 1 score^a^	0.160 ± 0.159	0.168 ± 0.170	0.263	0.295 ± 0.227	0.341 ± 0.256	0.005
BRCAPRO 2 score^a^	0.121 ± 0.119	0.121 ± 0.117	0.907	0.147 ± 0.175	0.162 ± 0.184	0.151
No. of patients with:	Mean^c^	Mean^c^		Mean^c^	Mean^c^	
BC^b^	2.31	2.31	0.746	2.34	2.36	0.636
OC^b^	0.28	0.32	0.153	0.31	0.33	0.165
BC and OC^b^	0.06	0.06		0.09	0.16	0.007
Bilateral BC^b^	0.42	0.43	0.415	0.42	0.47	0.169
BC in men^b^	0.04	0.04		0.06	0.08	0.161

^a^Paired *t*-test. ^b^Poisson log-linear regression model. ^c^Standard errors are in log scale, not shown

Further analysis of the separate components of the BRCAPRO model showed that frequencies from each of the parameters were higher in the recent pedigree, although only the increased number of patients with both breast and ovarian cancer reached statistical significance (mean ± SD, intake 0.09 ± 0.294, recent 0.16 ± 0.366, *p* = 0.007). Four females who developed both breast and ovarian cancer accounted for this difference. In two of them there were new events, one had already occurred before the intake but was not known to the index, and for the fourth case the date of diagnosis was unknown and therefore could not be distinguished if it was either a new or an unreported event. Conversely, the pathogenic variant detection probability in the wt families did not change significantly over time (Table 2).

In order to investigate the relative contributions of longer follow-up vs. higher cancer risks in the BRCAPRO scores of the most recent BRCAm pedigrees, we looked at the BRCAPRO scores of the BRCAm families to identify those which have changed with time, using the mean follow-up of the wild type families: 127 days as cut-off point. The comparison of the BRCAPROs between intake (0.443 ± 0.306) and follow-up at 127 days, (0.462 ± 0.311) showed no statistically significant difference (*p* = 0.168). Therefore differences occur after 127 days. Secondly,when comparing the most recent BRCAPRO scores of the wt families (0.290 ± 0.242) with the BRCAPRO scores of the BRCAm families at 127 days (0.462 ± 0.311) the difference remains statistically significant (*p* < 0.001).

## Discussion

In the current study we show that the pathogenic variant detection probability based on pedigree information increases significantly over time in families with a pathogenic variant, in contrast to families with wt *BRCA*. In particular, the number of women with both breast and ovarian cancer was significantly higher in the most recent pedigree. This indicates that, even if analyses are restricted to cancer history in first and second degree relatives, potential for bias needs to be considered when using family history information for the purposes of *BRCA* variant classification.

As expected, the mean BRCAPRO score was significantly higher in families with a pathogenic variant compared to wt families. This underscores the ability of the BRCAPRO model to distinguish between these two groups and is consistent with the results of previously published studies [5, 9, 10, 16]. A recent study from Germany including 7352 families confirmed that, compared with other risk prediction models, BRCAPRO and BOADICEA have the highest ability to discriminate between pathogenic variant carriers and non-carriers [16]. We specifically selected BRCApro over other possible tools since: (i) we hypothesized that potential bias between *BRCA* positive and negative pedigrees would be minimized since this tool only collects information on 1^st^ and 2^nd^ degree relatives; (ii) it was readily available in our clinic, and there was considerable user experience.

The follow-up time, i.e. the time between the date of the intake pedigree and the date of the last update was significantly longer in families with a pathogenic variant than in wt families, which is one of the factors that explains why pathogenic variant detection probabilities were different between the most recent and the intake pedigree in families with a pathogenic variant, as opposed to pedigrees with wt *BRCA*. This is in accordance with the fact that relatives from families with a pathogenic *BRCA* variant are offered predictive testing, which results in updating of pedigrees with newer or more accurate information. In contrast, relatives of probands with wt *BRCA* generally do not visit the genetics department and their pedigrees are not updated. The 127 days that on average had passed between intake and recent pedigree for wt families can largely be explained by the time that was needed for DNA testing and the time expended to obtain medical information from relatives.

In addition to longer follow-up, another factor contributing to the significant increase in pathogenic variant probabilities in the most recent pedigrees in the *BRCA* mutation group can be the underlying genetic cause, i.e. having a high penetrant cancer risk variant as opposed to the *BRCA* wt sequence families. The effect of the underlying genetic cause is shown by the fact that at the intake there was already a significant difference between both groups, and that this difference remained significant when comparing the groups at the shorter mean follow-up time observed for BRCA wt sequence families.

In conclusion, in this study we show that the *BRCA* pathogenic variant probability scores calculated in pedigrees from families with a pathogenic variant change over time, while pedigrees of wt families are less often updated and do not significantly change over time. These findings indicate that there is potential for bias which should be taken into account when using family history information in statistical models that assess the pathogenicity of *BRCA* variants, and possibly other high-risk cancer predisposition genes. We suggest that the simplest solution to avoid bias would be to use only information from intake pedigrees, or when these are not available, to exclude from analyses all cancer events that have occurred after the date of the intake.
